# Functional and Molecular Evidence for K_v_7 Channel Subtypes in Human Detrusor from Patients with and without Bladder Outflow Obstruction

**DOI:** 10.1371/journal.pone.0117350

**Published:** 2015-02-18

**Authors:** Julie Svalø, Majid Sheykhzade, Jørgen Nordling, Christina Matras, Pierre Bouchelouche

**Affiliations:** 1 Smooth Muscle Research Center, Department of Clinical Biochemistry, Copenhagen University Hospital, Koege, Denmark; 2 Department of Drug Design and Pharmacology, University of Copenhagen, Copenhagen, Denmark; 3 Department of Urology, Copenhagen University Hospital, Herlev, Denmark; 4 Department of Urology, Copenhagen University Hospital, Naestved, Denmark; University of Texas Health Science Center, UNITED STATES

## Abstract

The aim of the study was to investigate whether K_v_7 channels and their ancillary β-subunits, KCNE, are functionally expressed in the human urinary bladder. K_v_7 channels were examined at the molecular level and by functional studies using RT-qPCR and myography, respectively. We found mRNA expression of *KCNQ1, KCNQ3-KCNQ5* and *KCNE1-5* in the human urinary bladder from patients with normal bladder function (n = 7) and in patients with bladder outflow obstruction (n = 3). Interestingly, a 3.4-fold up-regulation of *KCNQ1* was observed in the latter. The K_v_7 channel subtype selective modulators, ML277 (activator of K_v_7.1 channels, 10 μM) and ML213 (activator of K_v_7.2, K_v_7.4, K_v_7.4/7.5 and K_v_7.5 channels, 10 μM), reduced the tone of 1 μM carbachol pre-constricted bladder strips. XE991 (blocker of K_v_7.1–7.5 channels, 10 μM) had opposing effects as it increased contractions achieved with 20 mM KPSS. Furthermore, we investigated if there is interplay between K_v_7 channels and β-adrenoceptors. Using cumulative additions of isoprenaline (β-adrenoceptor agonist) and forskolin (adenylyl cyclase activator) in combination with the K_v_7 channel activator and blocker, retigabine and XE991, we did not find interplay between K_v_7 channels and β-adrenoceptors in the human urinary bladder. The performed gene expression analysis combined with the organ bath studies imply that compounds that activate K_v_7 channels could be useful for treatment of overactive bladder syndrome.

## Introduction

The physiological roles of the urinary bladder are storage of urine and micturition. These processes are controlled both locally, by receptors in smooth muscle cells, urothelium and nerves, and centrally by higher brain levels [1]. Due to the complexity of the regulation of the detrusor, a number of diseases are linked to the lower urinary tract system. One of these diseases is overactive bladder (OAB) syndrome. It is a symptom complex that is defined by urgency, with or without urinary incontinence, nocturia and daytime urinary frequency, all symptoms that are related to the storage of urine [2]. Not only does OAB have a huge impact on the life-quality of the patients, but it also has a high prevalence, as many as 11.8% of the adult population suffers from OAB [3]. There are several causes of OAB, but in men, OAB is often associated with bladder outflow obstruction (BOO) due to benign prostatic hyperplasia [4]. For many years the first-in-line treatment for OAB has been muscarinic receptor antagonists. However, side effects, mainly dry mouth, leads to poor compliance and therefore there has been a search for novel targets. A recent approval of the β3-adrenoceptor agonist mirabegron by the FDA has improved the prospects for OAB patients [5].

During recent years, evidence have arisen, from studies on rat [6], guinea pig [7], pig [8] and from clinical trials with retigabine [9], that K_v_7 channels are key regulators of urinary bladder contractility. More specifically, it has been shown that retigabine increases micturition volume in rats [6], reduces tone and contractility of rat and pig detrusor strips [10], and enhances K_v_7 potassium currents in isolated detrusor smooth muscle cells [11] and interstitial cells [7]. There are five different subtypes of the K_v_7 channels, K_v_7.1–7.5 [12] and the protein is encoded by the *KCNQ* gene. The potassium channels are formed as either homo- or hetero-tetrameric combinations of K_v_7.2/7.3, K_v_7.3/K_v_7.5 or K_v_7.4/K_v_7.5 alpha subunits. K_v_7 channels are voltage-gated potassium channels, but they can be positively modulated by pharmacological compounds. The channels can associate with ancillary β subunits, encoded by *KCNE1–5* genes. KCNE proteins are accessory subunits to multimeric ion channels and thus important regulators of both the resting membrane potential and cellular excitability [13].

According to numerous studies, K_v_7 channels are distributed throughout the body, both in the cardiovascular system and in non-vascular smooth muscle cells [14]. Though the transcription of K_v_7.2 and K_v_7.3 channel has been described in both sympathetic [15,16], sensory [17–19] and hippocampal neurons [20], and K_v_7.4 and K_v_7.5 in the vasculature [21,22]; only a few studies have confirmed these findings in human tissues. More specifically, K_v_7 channels have only been studied in detail in human myometrium [23], mesenteric arteries and arteries from visceral adipose tissue [24] and airway smooth muscle [25]. For this reason, to increase the knowledge of K_v_7 channel modulation for the treatment of human diseases, there is a need for studies on human tissue. More specifically, there is a need to know which subtypes of K_v_7 channels that are expressed and functional in the human urinary bladder. This information, along with our knowledge from other organs, will assist us to predict side effects of K_v_7 channel modulators.

In the present study, we focused on K_v_7 channels in human bladder biopsies as these, to our knowledge, has not been studied elsewhere. We analysed the molecular expression of *KCNQ* and *KCNE* in the urinary bladder and compared these to the corresponding levels in BOO patients as patients with BOO are more prone to develop OAB compared to patients without BOO. The molecular studies were accompanied by pharmacological studies using the activators retigabine (K_v_7.2–7.5, D23129, N-(2-amino-4–(4-flurobenzylamino)-phenyl) carbamic acid ethyl ester), ML213 (K_v_7.2, K_v_7.4, K_v_7.4/7.5 and K_v_7.5, N-Mesitylbicyclo[2.2.1]heptane-2-carboxamide) [26,27], and the more recently developed compound ML277 (K_v_7.1, (R)-N-(4-(4-methoxyphenyl)thiazol-2-yl)-1-tosylpiperidine-2-carboxamide) [28]. To inhibit K_v_7 channels, we have used the pan-selective compound XE991 (K_v_7.1–7.5, 10,10-bis(4-pyridinyl-methyl)-9(10H)-anthracenone). The functional studies were performed on bladder strips either pre-constricted with muscarinic receptor agonist carbachol or depolarised with the buffer solution containing 20 mM or 40 mM K^+^ to elucidate the efficacy of K_v_7 channel modulation of isolated detrusor smooth muscle strips was affected by varying the extracellular K^+^ concentration thus confirming K^+^-channel selectivity. The β3-adrenoceptors have been shown to couple to different potassium channels in various tissues [29], and it has been published, that reduced K_v_7.4 channel function leads to impairment of β-adrenoceptor mediated relaxation in α1-adrenoceptor pre-constricted renal arteries in rats [22]. K_v_7.1 channels have been shown to have an A-kinase anchoring protein (AKAP) binding site to which protein kinase A can be recruited and mediate phosphorylation of the channel [30,31]. Furthermore, phosphorylation of K_v_7.1, K_v_7.2/K_v_7.3 and K_v_7.4 channels by protein kinase A has been shown to significantly alter the currents of the channels [32–34]. Therefore, to fully understand the β-adrenoceptor mediated relaxations in different tissues, more studies on the mechanism of action are needed [35]. To further increase our understanding of the mechanism of β-adrenoceptor mediated relaxation, we tested if β-receptor and K_v_7 channel mediated relaxation were two independent mechanisms or if they were interrelated [22].

## Materials and Methods

### Ethics

Patients were recruited from the department of Urology at Herlev, Naestved and Skejby Hospitals and the experiments were performed at the Smooth Muscle Research Center, Copenhagen University Hospital at Koege. The study has been approved by the Copenhagen County Ethics Committee (SJ-219).

Detrusor tissue was obtained, with informed written consent, from patients with normal bladder function undergoing cystoscopy (7 patients; mean age 63; range 45–73 years, International Prostate Symptom Scores (I-PSS): 2.3 ± 0.7, for RT-qPCR analysis) or cystectomy (15 patients; mean age 68; range 56–76 years, I-PSS data for 6 out of 15 patients: 8.8 ± 1.9, no preoperative symptoms of overactive bladder, for myography). Detrusor specimens were obtained during surgery with special care taken to remove the samples from a non-tumour infiltrating area. Detrusor specimens from BOO patients were obtained from those undergoing transurethral resection of the prostate due to benign prostatic hyperplasia (3 patients, mean age of 66 years, range 62–75 years, I-PSS 22.3 ± 4.1, for RT-qPCR analysis). All BOO patients were urodynamically characterised.

Detrusor specimens to be used for organ bath studies were kept in 4°C Tyrode’s salt solution (Sigma-Aldrich Denmark A/S, Broendby, Denmark) with 20 mM HEPES (Life Technologies Europe B.V., Naerum, Denmark), (pH at 7.4) and transported immediately to the Smooth Muscle Research Center at Koege Hospital. Biopsies for RT-qPCR analysis were kept in RNAlater (Qiagen Denmark, Copenhagen, Denmark), stored at 4°C overnight, and then frozen at -20°C until further processing.

### RNA extraction, cDNA synthesis and RT-qPCR analysis

The methods have formerly been described by Rosenbaum et al. [36] with modifications. Tissue biopsies were homogenised in liquid nitrogen, and in 1 ml TriZol (Life Technologies Europe B.V., Naerum, Denmark), using a rotor-stator homogeniser. Total RNA was extracted from (50–150 mg) homogenised tissue using the Trizol method (manufacturer’s instruction), including bromochloropropane (VWR, Herlev, Denmark) for phase-separation. The quantity and quality of extracted RNA was checked by gel electrophoresis with a Bioanalyzer 2100 (Agilent Technologies, Hoersholm, Denmark). Samples had a RIN value of 5.6–8.9 which ensured a high RNA integrity. RNA (2.5–10 μg) samples were purified by DNase treatment in order to remove contaminating genomic DNA. This was done using an RNase-free DNase kit according to the manufacturer’s instructions for DNA removal (50 μl reaction) (Ambion, Life Technologies Europe B.V., Naerum, Denmark). The quantity and quality of DNase treated RNA was checked by gel electrophoresis using a Bioanalyzer 2100.

RNA was reversely transcribed into complementary DNA, (cDNA) with 7.5 μl oligo(dT) primers (100 ng/μl), and reverse transcriptase (2.5 μl), in a 50 μl reaction volume from 1700 or 2000 ng DNase treated RNA. This was done using the Affinity Script qPCR cDNA synthesis kit (Stratagene, AH Diagnostics, Aarhus, Denmark) as per the manufacturer’s instructions. Cycling parameters were 5 minutes at 25°C; 15 minutes at 42°C; 5 minutes at 95°C; and 30 minutes at 25°C using a Mx3000P QPCR system (Stratagene, AH Diagnostics, Aarhus, Denmark). cDNA was stored at -20°C until further processing.

Primers for the genes of interest, *KCNQ1-5* and *KCNE1-5*, and for the reference genes, *GAPDH* and *ACTB*, were designed using Primer3 (ncbi). Primer properties were determined by Oligo Analyzer (IDT) software (Table 1). The primers were synthesised and purified by MALDI-TOF from TAG-Copenhagen (Denmark). The primers were designed to match all known splice variants of the given gene. To ensure primer specificity a BLAST search against the NCBI database was performed, and a melting curve analysis ensured that only one product was amplified. Primers for the additional reference genes *B2M*, *YWHAZ*, *SF3A1* and *18S* were included in a geNorm REF gene kit (PrimerDesign Ltd, Southampton, UK), which included the qbasePLUS software license.

**Table 1:** RT-qPCR primer pair sequences, amplicon and standard curve data.

Gene	Primer sequence (+) Sense, (-) antisense	Genebank Accession number	Region spanned	Amplicon bp	Efficiency (%)	R^2^
*β-actin*	(+) TCCCCCAAAGTTCACAATGTGG	NM_0001101	1399–1496	98	92.7	0.996
	(-) GGGACTTCCTGTAACAACGCAT					
*GAPDH*	(+) CAGTCAGCCGCATCTTCTTTTG	NM_002046	49–148	100	94.4	0.996
	(-) GCCCAATACGACCAAATCCGTT					
*KCNQ1*	(+) AGAACCAACAGCTTCGCCGAGG	NM_000218	1549–1684	136	81.7	0.986
	(-) TGGCCACAAAGTACTGCATGCG					
*KCNQ2*	(+) CACAGGCAGAAGCACTTTGAGA	NM_172107	1147–1280	134	79.3	0.995
	(-) GTGACCGTTCGCTCGTAGTAC					
*KCNQ3*	(+) TGCCGCCACCTTTTCCTTAATT	NM_004519	1221–1333	113	79.8	0.995
	(-) TCTCAAAGTGCTTCTGACGGTG					
*KCNQ4*	(+) ATCTCTGAGACTCAAACCCCGC	NM_004700	1561–1676	116	103.2	0.991
	(-) TCTTCACAGCAGGCATGATGTC					
*KCNQ5*	(+) CTTGGCTGGGAAGATTGCTTTC	NM_019842	1309–1440	132	86.1	0.987
	(-) TCTCAAAGTGTTTCTGGCGGTG					
*KCNE1*	(+)-GTGATCTTTGCCCCACCCGA	NM_001127670	2768–2870	103	84.1	0.985
	(-) TGAGACGTGGCAGGCATAGC					
*KCNE2*	(+)-GAGAACATTGGTGCGGCTGG	NM_172201	474–575	102	87.7	0.958
	(-)-TCGTGGCACTGGCATCTCTTC					
*KCNE3*	(+)-CAAGACACCTGGGGATTGCGT	NM_005472	687–841	135	93.9	0.976
	(-)-GCACAAGGCTTCGGTCTACCA					
*KCNE4*	(+)-GCTTCCCCTTTGGCCGGTTA	NM_080671	2023–2129	107	102.6	0.995
	(-)-TGGGTTGGTAGCTTGCCCTG					
*KCNE5*	(+)-AGTGCTCAACGGGGAGGGAA	NM_012282	1179–1250	72	78.5	0.994
	(-)-GGGATGAAAACTGGGGAGGCA					

RT-qPCR was performed using the Brilliant SYBR Green QPCR Master Mix and Mx3000P QPCR system (both from AH Diagnostics, Aarhus, Denmark). Reactions were carried out in 12.5 μl reaction volumes containing 20 ng of cDNA; 6.25 μl brilliant SYBR green mastermix; 0.25μl (10 pmol/μl) of each primer; and 6.25 μl RNase-free H_2_O. Amplification was detected using SYBR green fluorescent dye with the following amplification conditions: i) one 10 minutes pre-amplification cycle at 95°C; ii) 40 amplification cycles including 30 sec (denaturation) at 95°C; 60 sec (annealing) at 60°C; and 60 sec (extension) at 72°C; iii) 60 sec end-denaturation cycle at 95°C, followed by increasing the temperature from 55°C to 95°C with continuous reading of fluorescence data (dissociation curve).

Standard curves were performed for the stably expressed reference genes, and for the genes of interest (*KCNQ1-5* and *KCNE1-5*), by 2-fold serial dilution of a cDNA pool of the positive controls brain and heart (Clontech, BioNordika, Herlev, Denmark) (0.625–80 ng) (for *KCNQ4* and *KCNE4* the concentration was 0.59–300 ng).

Controls with no template- and no reverse transcription (RT) were performed for each sample and primer pair when appropriate to control for contamination or primer-dimers. A ΔCq > 10 between sample and the control with no RT ensured that the samples were not contaminated by any form of genomic DNA. All reactions were carried out in triplicate, in regard to both samples and standard curves. Cq values were calculated from the exponential phase of amplification when crossing threshold using MxPRO 4.1 software (AH Diagnostics, Aarhus, Denmark). Cq values were transformed into relative quantities using the gene-specific PCR amplification efficiency calculated by geNormPlus software (qbasePLUS, Biogazelle, PrimerDesign Ltd, Southampton, UK). A geometric mean of the two stable reference genes was employed as the normalisation factor of the reference genes in order to calculate the relative expression level of *KCNQ1-5* and *KCNE1-5* within the two patient groups. While amplicons were sequenced in order to ensure *KCNQ* and *KCNE* primer specificity (Beckman Coulter Genomics, Takeley, UK), a BLAST search against the NCBI database confirmed that only the gene of interest was amplified. All graphical presentations were prepared using GraphPad Prism 5.04 (San Diego, California, USA). Values were presented as mean (95% CI), and group differences were analysed using the Mann-Whitney test.

### Organ bath studies

Retigabine and forskolin were purchased from Alamone Labs (Jerusalem, Israel) whereas XE991 was kindly provided by NeuroSearch (Ballerup, Denmark), and ML213 and ML277 were kindly provided by Vanderbilt University (Nashville, TN, USA (MLCPN probes)). Papaverine and isoprenaline were from Sigma-Aldrich (St. Louis, MO, USA) whereas carbachol and tetrodotoxin (TTX) were purchased from Tocris (VWR international, Herlev, Denmark) and Ascent Scientific (Cambridge, UK), respectively. All stock solutions (10^–2^ M), except for isoprenaline (dissolved in water), were prepared in 100% DMSO (Merck, VWR international, Herlev, Denmark). Further dilutions were prepared in physiological saline solution (PSS) (NaCl 118.99 mM, KCl 4.69 mM, MgSO_4_•7H_2_O 2.40 mM, KH_2_PO_4_ 1.18 mM, glucose 6.06 mM, NaHCO_3_ 25 mM, CaCl_2_ 1.6 mM, EDTA 0.025 mM, pH 7.4) just before experiments.

Upon arrival the tissue was immediately placed in cold oxygenated PSS. The detrusor was separated from the urothelium under a stereomicroscope. The detrusor was then cut into strips, each 3 mm in length and 1 mm in width, weighing 3.16 ± 0.06 mg (n = 226) and kept on ice. The strips were then mounted in the organ bath (1 ml) of a myograph (700MO; Danish Myo Technology (DMT), Denmark), kept at 37°C and bubbled with a gas mixture of 95% oxygen and 5% carbon dioxide to a pH of 7.4 in PSS-buffer. The digital output was transformed using a powerlab (AD instruments, DMT, Aarhus, Denmark).

The strips were stretched by 2.5 mm to a total length of 4.09 ± 0.03 mm (n = 226) during 30 minutes followed by an equilibration period of 60 minutes resulting in a mean baseline tone of 6.88 ± 0.63 mN (n = 226), in this period the bath solution was changed at 15 minutes intervals.

Strips were stimulated with 20 mM KPSS, 40 mM KPSS or 1 μM carbachol producing a mean tone of 11.85 ± 1.37 mN (n = 47), 18.84 ± 1.67 mN (n = 98) and 31.68 ± 2.14 mN (n = 81), respectively. When contractions were stable, concentration-responses of the effect of retigabine (0.01–30 μM) and ML213 (0.01–30 μM) were evaluated on 1 μM carbachol pre-constricted tissue at a 10 minutes dosing-interval. Alternatively, strips pre-constricted by KPSS (20 mM or 40 mM) or carbachol (1μM) were treated with retigabine (10 μM), ML213 (10 μM), ML277 (10 μM), XE991 (10 μM) or vehicle control for 15 minutes. This allowed for the evaluation of K_v_7 channel mediated effects. To study the involvement of Kv7 channels in the relaxation produced by stimulation of β-adrenoceptors and activation of adenylyl cyclase activity, the strips pre-treated with retigabine, XE991 or vehicle were treated with cumulative addition of isoprenaline (0.001–3 μM), forskolin (0.01–30 μM), or time-dependent vehicle-control at a 10 minutes dosing-interval. On tissue pre-constricted by 20 mM KPSS only a single addition of isoprenaline (0.1 μM), forskolin (1 μM) or time-dependent vehicle-control was added for 10 minutes. Maximal relaxation was induced by 100 μM papaverine whereas strips viability was assessed by stimulations of the detrusor strip with a buffer containing 125 mM K^+^ (KPSS). KPSS was similar to PSS except that NaCl was exchanged with KCl on an equimolar basis.

All values were normalised to the constriction achieved after the addition of the constriction agent before addition of compound. The mean tone was calculated, using LabChart Pro 7.3.7 software (AD instruments, DMT, Aarhus, Denmark), from an average of the last 2 minutes trace just before addition of the next concentration of drug. Graphic presentations were prepared using GraphPad Prism 5.04 (GraphPad Software, San Diego California, USA), E_max_ values were calculated as maximum obtained effects compared to time-dependent vehicle-controls. Cumulative additions of compound were analysed using two-way repeated measures ANOVA followed by Bonferroni post-hoc test. Differences in mean tone between compound and vehicle control was analysed by unpaired *t*-test. The number of patients is denoted by *n* and data are presented as mean values ± S.E.M.

## Results

### Molecular identification of KCNQ and KCNE subtypes

Before running the RT-qPCR analysis of *KCNQ1-5* and *KCNE1-5* gene transcript levels, a geNorm analysis was performed to determine which genes were most appropriate to use for normalisation. The analysis showed that, when analysing bladder samples, the most stably expressed genes were as follows: *GAPDH* > *ACTB* > *TOP1* > *B2M* > *SF3A1* > *18S*. Furthermore, the analysis depicted that the inclusion of the invariant controls GAPDH and ACTB would be appropriate for normalisation purposes.

An M-value of 0.327 ± 0.11 (n = 10) was found when analysing bladder samples from the two patient groups. An M-value below 0.5 is considered to be a high reference target stability [37].

**Fig 1:**
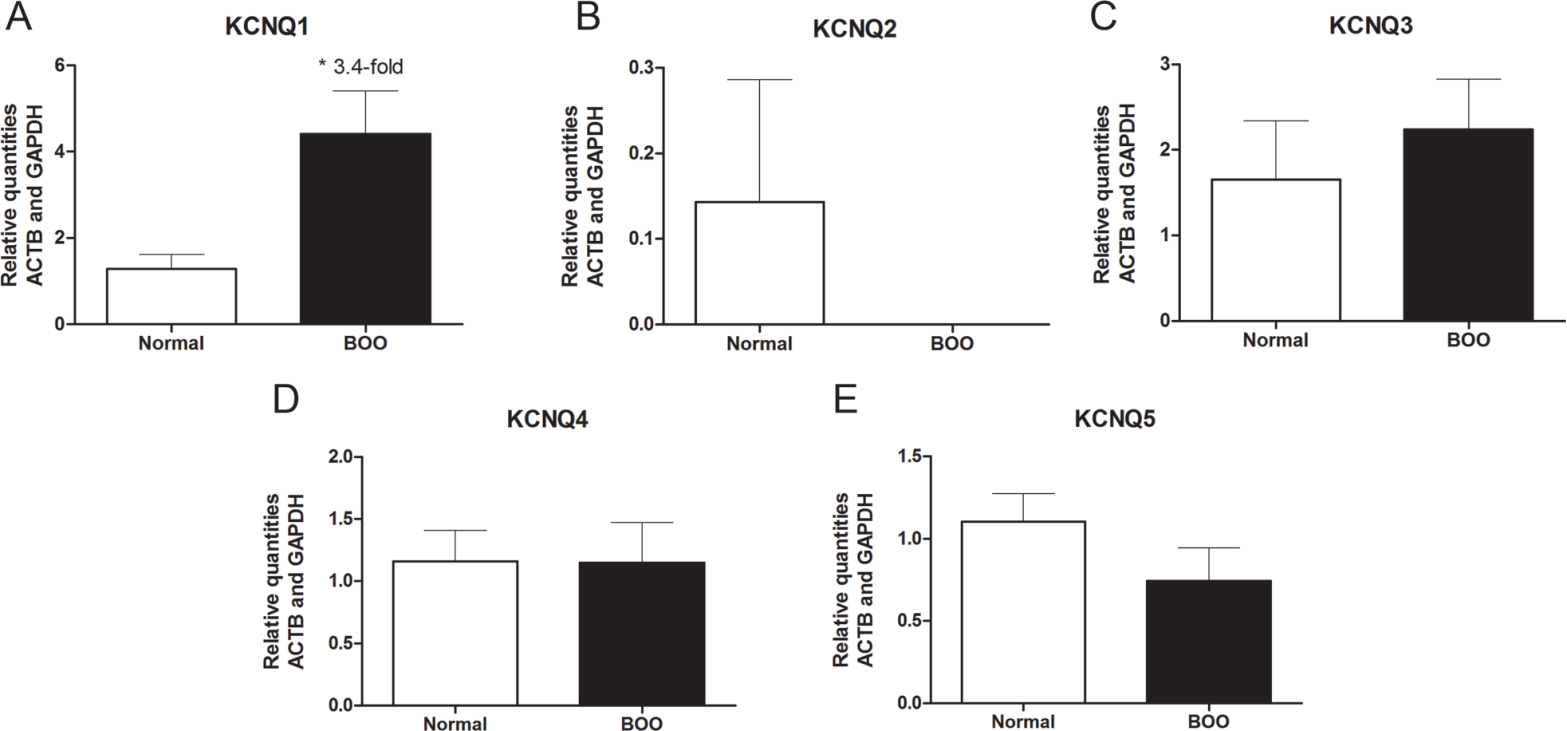
mRNA transcript levels of KCNQ in human urinary bladder. *KCNQ1-5* mRNA transcript levels were determined in bladder tissue from patients with normal bladder function (normal, n = 7) and in bladder tissue from patients with bladder outflow obstruction (BOO, n = 3). * *P*<0.05, compared to normal bladder tissue. Normalisations were performed by the inclusion of the invariant controls ACTB and GAPDH.

**Fig 2:**
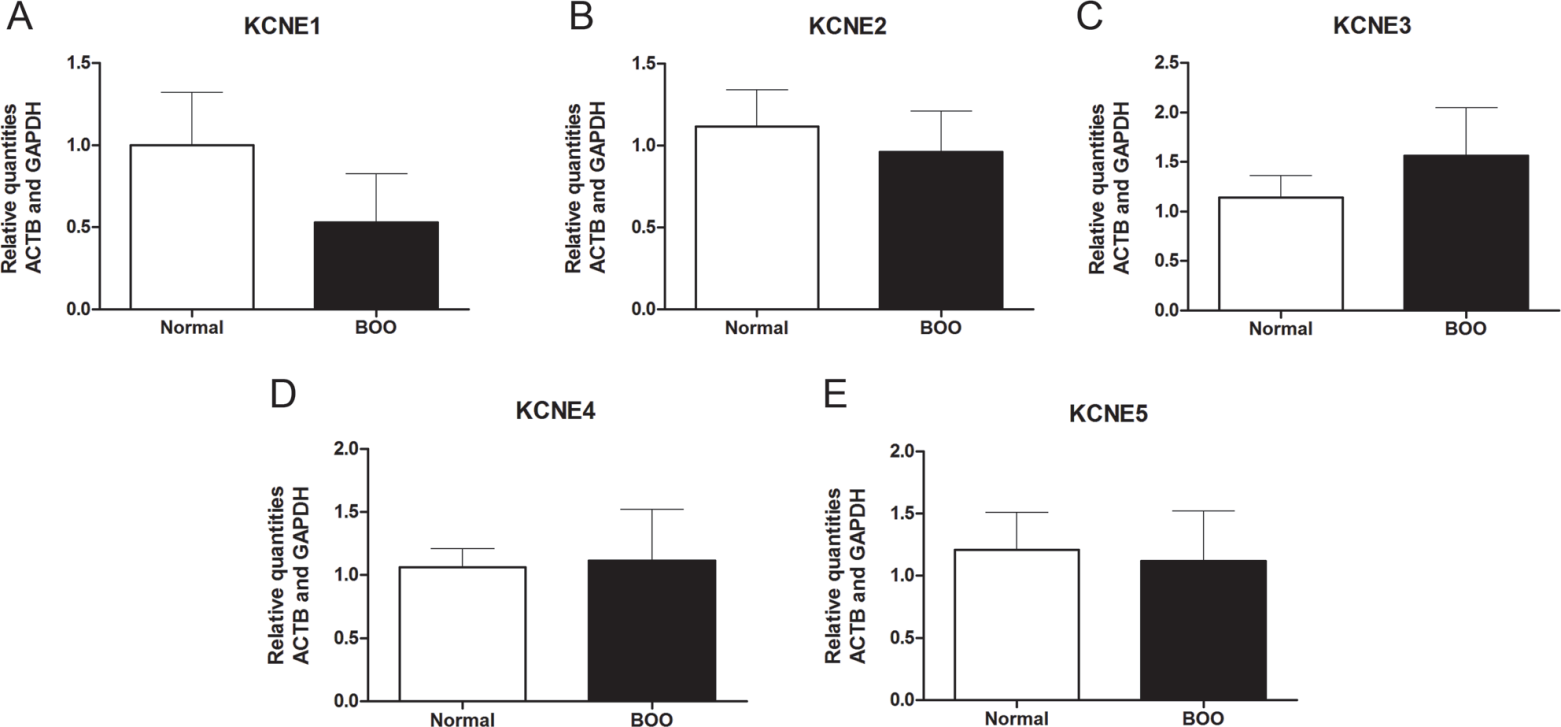
All known subtypes of the *KCNE* gene are expressed in human bladder. *KCNE1-5* mRNA transcript levels were determined in bladder tissue from patients with normal bladder function (normal, n = 7), in bladder tissue from patients with bladder outflow obstruction (BOO, n = 3). Normalisations were performed by the inclusion of the invariant controls ACTB and GAPDH.

*KCNQ1-5* and *KCNE1-5* mRNA transcripts were evaluated in bladder tissue from patients with normal bladder function (n = 7) as well as in patients with bladder outflow obstruction due to prostatic hyperplasia (n = 3). In the present study, all *KCNQ* and *KCNE* subtypes were detected in human urinary bladder biopsies, except for *KCNQ2* in which only one patient (normal group) had a level of *KCNQ2* that was above the detection limit (Cq<35). *KCNE4* and *KCNQ4* were the most abundantly expressed genes depicted by raw Cq values. The relative quantities of mRNA transcripts of *KCNQ2-5* and *KCNE1-5* in bladder did not differ between the two patient groups (Figs. 1 and 2). However, the level of *KCNQ1* transcript was 3.4-fold higher in patients with BOO compared to patients with normal bladder function (*P* = 0.017, Fig. 1A).

### Functional effect of K_v_7 channel modulators on human detrusor strips

**Fig 3:**
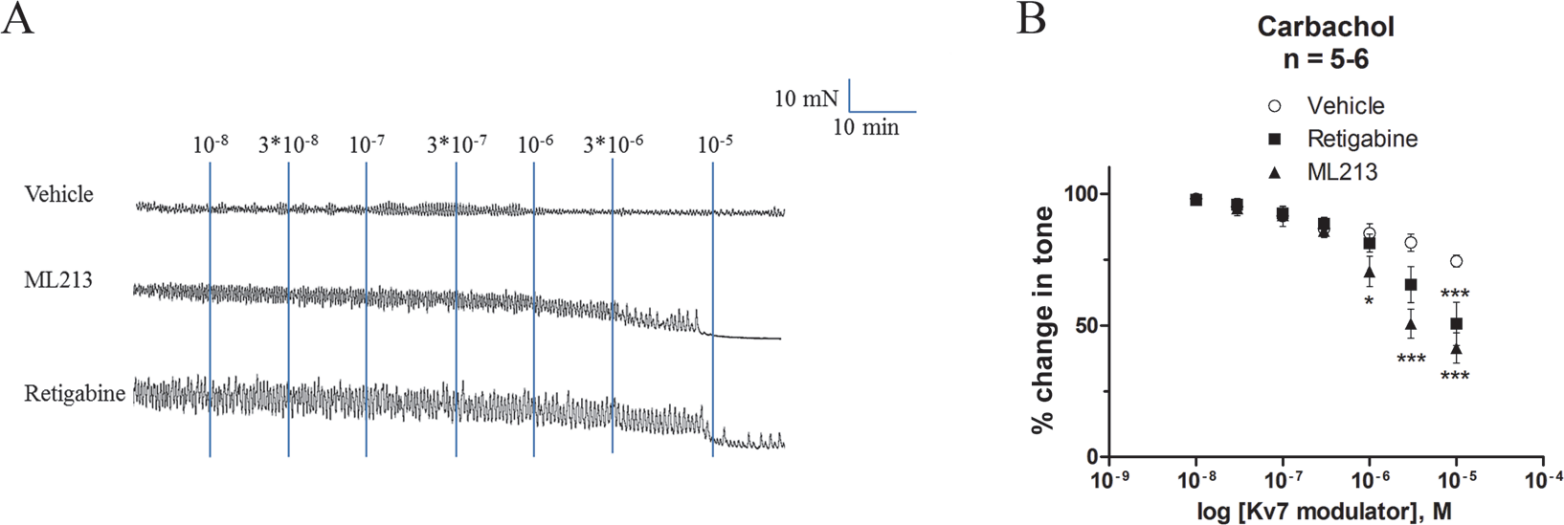
Retigabine and ML213 decrease the constriction tone of human detrusor strips. (A) The original DSM tension recordings illustrate the relaxatory effect of retigabine and ML213, which are K_v_7.2–7.5 and K_v_7.2/7.4/7.5 channel activators, respectively. (B) Cumulative concentration-responses for retigabine, ML213 and time-matched vehicle responses (< 0.1% DMSO). Initial tone was achieved by pre-constriction with 1 μM carbachol. Retigabine decreased mean tone with a lower efficacy compared to ML213. *P<0.05, ***P<0.001, significantly different from vehicle control.

To study the importance of K_v_7 channels on urinary bladder contractility several K_v_7 channel modulators were tested using different pre-constricting agents. The positive modulator retigabine (K_v_7.2–7.5) decreased the constriction achieved by 20 mM KPSS (*P* = 0.030), 40 mM KPSS (*P* = 0.014) and 1 μM carbachol (*P* = 0.001), compared to vehicle control (Table 2). The concentration-response of retigabine was analysed at the tone induced with 1 μM carbachol, the effect of retigabine differed significantly (*P*<0.001) from that of vehicle control above 3 μM, and retigabine reduced mean tone to 50.67 ± 8.27% of the initial tone with carbachol (Fig. 3). The vehicle responses declined gradually over time being significantly reduced at and above a concentration of 0.01% DMSO (*P*<0.05). To elaborate on the effects of retigabine, the more selective compounds ML277 and ML213 were tested under the same experimental conditions. ML277, which activates K_v_7.1 channels, reduced tone induced by the pre-constrictor agents 1 μM carbachol and 40 mM KPSS, whereas ML213, which is known to activate both K_v_7.2 and K_v_7.4 channels, but also K_v_7.4/7.5 and K_v_7.5 channels, only affected tone induced by 1 μM carbachol (Table 2). ML213 concentration-dependently reduced the carbachol evoked response to 41.41 ± 5.71% of the initial pre-constriction tone achieved by carbachol, thereby being more efficacious than retigabine (*P*<0.05) (Fig. 3).

**Table 2:** Reduction in mean tone by Kv7 channel modulators (10 μM) in human DSM isolated strips.

Stimuli	Modulator	Target	Mean tone(%)	*P*
1μM carbachol	Retigabine	Pos. Kv7.2–7.5	20.76 ± 4.97	** (6)
	ML213	Pos. Kv7.2/7.4	32.93 ± 6.63	*** (6)
	ML277	Pos. Kv7.1	13.11 ± 3.95	* (4)
	XE991	Neg. Kv7.1–7.5	- 4.23 ± 3.46	(7)
40mM KPSS	Retigabine	Pos. Kv7.2–7.5	8.08 ± 2.90	* (7)
	ML213	Pos. Kv7.2/7.4	1.45 ± 7.67	(7)
	ML277	Pos. Kv7.1	19.81 ± 4.13	** (4)
	XE991	Neg. Kv7.1–7.5	2.18 ± 2.51	(10)
20mM KPSS	Retigabine	Pos. Kv7.2–7.5	19.34 ± 7.34	* (4)
	ML213	Pos. Kv7.2/7.4	13.81 ± 12.64	(4)
	ML277	Pos. Kv7.1	6.34 ± 6.10	(4)
	XE991	Neg. Kv7.1–7.5	-43.89 ± 19.01	* (5)

Mean tone is reported in comparison to time-matched vehicle control (mean difference ± SEM%). Positive and negative modulators are denoted by Pos. and Neg., respectively. Each data point is n = 4–10 patients which is presented in parenthesis,

*P<0.05,

**P<0.01,

***P<0.001 vs. vehicle control and is tested by *t*-test.

To investigate whether K_v_7 channels are important regulators of urinary bladder quiescence, the pan-selective K_v_7 channel blocker XE991 was applied to the muscle strips. XE991 increased the pre-constriction tone achieved with 20 mM KPSS by 43.89 ± 19.01% compared to control (*P* = 0.043) whereas XE991 had no effect on the pre-constriction tone achieved by 40 mM KPSS (*P* = 0.397) or 1 μM carbachol (*P* = 0.243) (Table 2).

### Effect of isoprenaline and forskolin on detrusor contractility

**Fig 4:**
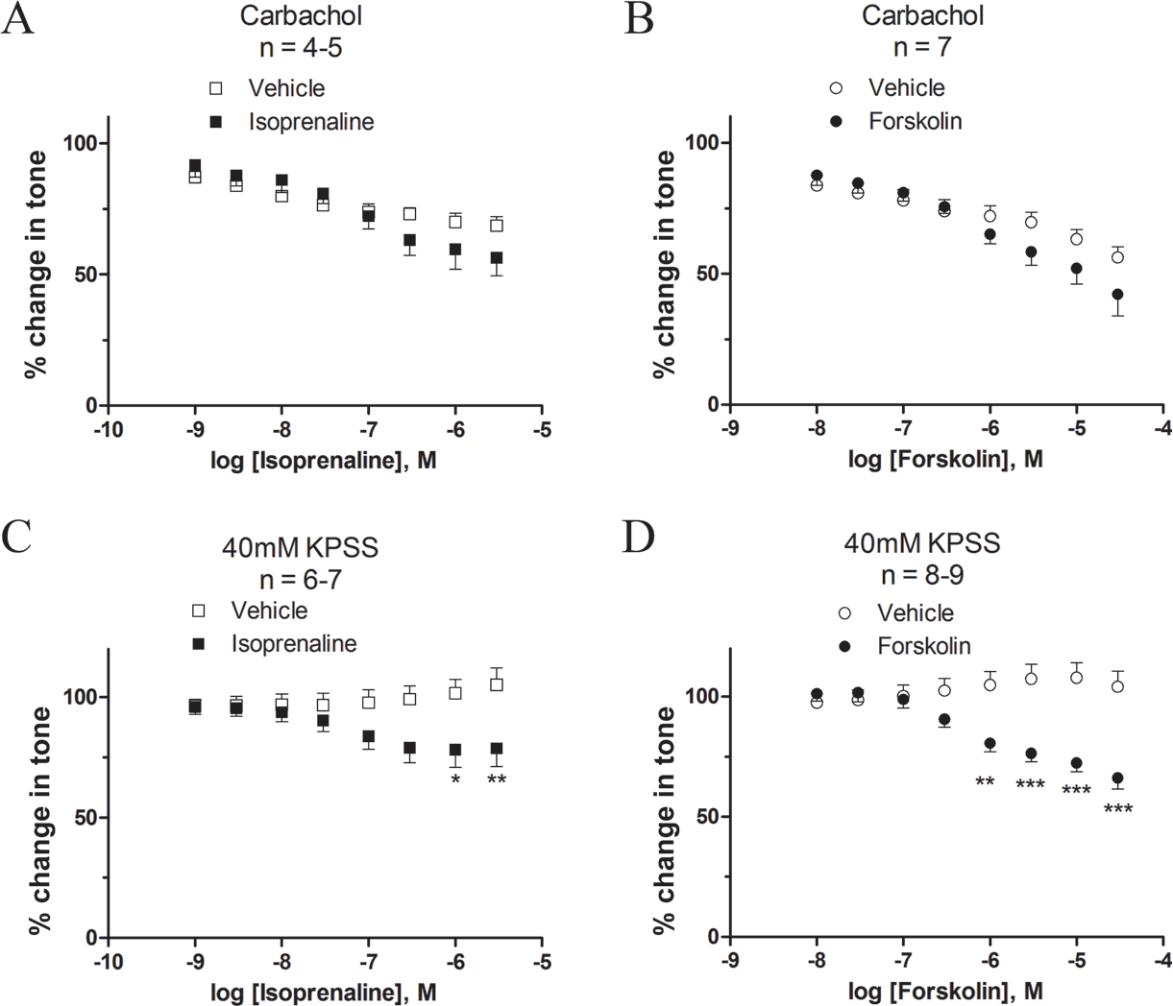
Effect of isoprenaline and forskolin in human DSM at 1 μM carbachol- and 40 mM KPSS-induced contractions. Cumulative concentration-responses were induced with isoprenaline and forskolin. Initial tone was achieved by pre-constriction with 1 μM carbachol (A and B) and 40 mM KPSS (C and D) bladder strips. *P<0.05, **P<0.01, ***P<0.001, significantly different from vehicle control. The vehicles corresponded to water (< 0.2%)(A and C) and DMSO (< 0.3%)(B and D) diluted in PSS.

Pre-constriction of urinary bladder strips with 1 μM carbachol was followed by cumulative additions of isoprenaline (Fig. 4A and Table 3). Forskolin also relaxed carbachol-contracted strips, with maximal efficacy equivalent to isoprenaline (*P* = 0.25) (Fig. 4B and Table 3). When evaluating the effects of isoprenaline and forskolin on 40 mM KPSS-depolarised strips, both isoprenaline (Fig. 4C) and forskolin (Fig. 4D) concentration-dependently reduced the mean tone of detrusor strips compared to the time-dependent vehicle-control, however; forskolin being the most efficacious compound (*P*<0.01). The maximum reduction in the mean tone by both isoprenaline and forskolin compared to vehicle effect was at least twice as large in 40 mM KPSS compared to 1 μM carbachol pre-constricted strips, though the difference was not statistically significant (*P* = 0.46 and *P* = 0.07, respectively). The time-matched vehicle controls declined gradually over time in 1 μM carbachol pre-constricted tissues, but not when the tissues were pre-constricted with 40 mM KPSS (Fig. 4).

**Table 3:** Reduction in mean tone by isoprenaline and forskolin with or without pre-incubation with K_v_7 channel modulators (10μM) in human DSM isolated strips.

Stimuli	Modulator	Target	Mean tone(%)	P
1μM carbachol	Isoprenaline	Beta-AR agonist	12.21 ± 8.34	0.19
	+ retigabine	Pos. Kv7.2–7.5	17.97 ± 5.79	0.47^§^
	+ XE991	Neg. Kv7.1–7.5	6.91 ± 7.32	0.74^§^
	Forskolin	AC activator	13.97 ± 9.26	0.16
	+ XE991	Neg. Kv7.1–7.5	8.36 ± 6.00	0.67^#^
40mM KPSS	Isoprenaline	Beta-AR agonist	26.45 ± 10.47	*
	+ retigabine	Pos. Kv7.2–7.5	26.30 ± 4.28	0.97^§^
	+ XE991	Neg. Kv7.1–7.5	28.61 ± 7.89	0.80^§^
	Forskolin	AC activator	38.03 ± 8.10	***
	+ XE991	Neg. Kv7.1–7.5	34.08 ± 11.35	0.72^#^

Mean tone is reported as mean difference ± SEM% in comparison to time-matched vehicle-control at 30 μM forskolin or 3 μM isoprenaline. Positive and negative modulators are denoted by Pos. and Neg., respectively, and AC stands for adenylyl cyclase. Each datapoint is n = 4–9 patients. Statistically significant differences were evaluated by *t*-test,

*P<0.05,

***P<0.001 compared to vehicle control,

^§^ compared to isoprenaline or

^#^ compared to forskolin.

### Mechanism of β-adrenoceptor-mediated relaxation

**Fig 5:**
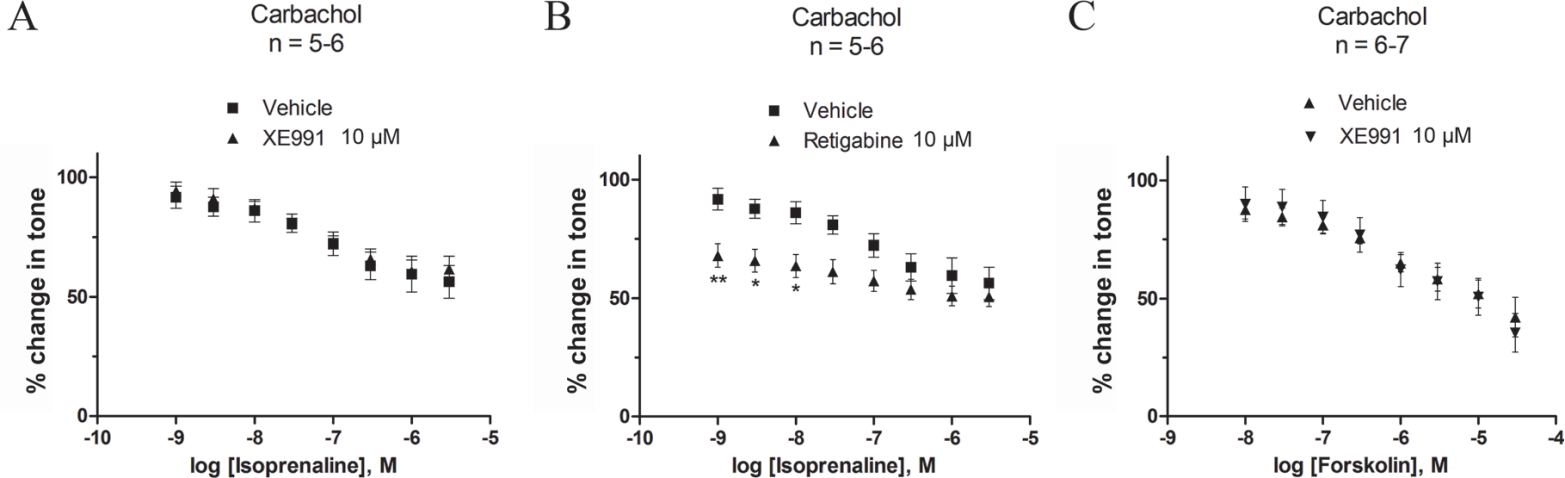
Modulation of K_v_7 channels does not affect Gs mediated relaxation at 1 μM carbachol-induced contractility. Cumulative concentrations-responses for isoprenaline (A and B) and forskolin (C) were produced with pre-incubation of 10 μM XE991 (A and C), 10 μM retigabine (B) or vehicle control (0.1% DMSO). Initial tone was achieved by pre-constriction with 1 μM carbachol. *P<0.05, **P<0.01, significantly different from isoprenaline. When pre-constricting the detrusor strips by 40 mM KPSS neither of the treatments with K_v_7 channel modulators (retigabine and XE991) changed the response to isoprenaline or forskolin at any concentration tested (Fig. 6).

In order to study whether the Gs pathway was coupled to K_v_7 channels, we analysed the effect of activating and blocking the K_v_7 channels by retigabine (Fig. 5B) and XE991 (Fig. 5A and C), respectively, before cumulative additions with isoprenaline (Fig. 5A-B) and forskolin (Fig. 5C). This was tested in strips pre-constricted with 1 μM carbachol. The addition of XE991 (10 μM) had no effect on the efficacy of isoprenaline or forskolin (Table 3). At 1 nM of isoprenaline, retigabine (10 μM) reduced tone by 23.69% as compared to the effect of isoprenaline alone (*P*<0.01). This is equivalent to the effect of retigabine (10 μM) on 1 μM carbachol pre-constricted detrusor strips described in Table 2.

**Fig 6:**
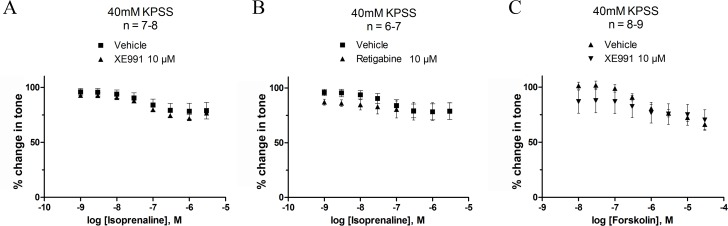
Modulation of K_v_7 channels does not affect Gs mediated relaxation at 40 mM KPSS-induced contractility. Cumulative concentrations-responses for isoprenaline (A and B) and forskolin (C) were produced with pre-incubation of 10 μM XE991 (A and C), 10 μM retigabine (B) or vehicle control (0.1% DMSO). Initial tone was achieved by pre-constriction with 40 mM KPSS. Neither of the K_v_7 channel modulators affected the response to isoprenaline or forskolin.

**Fig 7:**
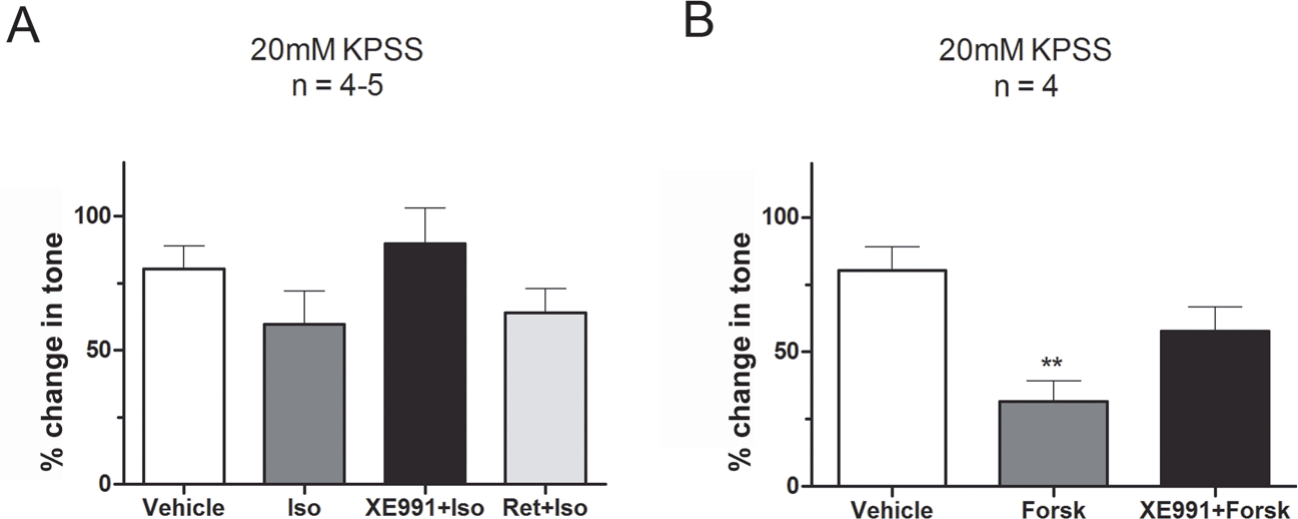
At low depolarisation K_v_7 channel inhibition reduces the response to forskolin. Strips pre-constricted with 20 mM KPSS were treated with either 0.1 μM isoprenaline (A) or 1 μM forskolin (B). Pre-treatment with K_v_7 channel modulators revealed a reduction in response to isoprenaline and forskolin when blocking K_v_7 channels by XE991 (10 μM) whereas retigabine (10μM) did not affect the response to isoprenaline. Pre-treatment with vehicle control (0.1% DMSO). **P<0.01, significantly different from vehicle control.

To further investigate the role of the beta-adrenergic pathway, we added either retigabine (10 μM) or XE991 (10 μM), on the top of 20 mM KPSS pre-constricted strips before addition of isoprenaline (0.1 μM) (Fig. 7A and B) or forskolin (1 μM) (Fig. 7C). Isoprenaline reduced the mean tone by 20.60 ± 16.01% compared to vehicle control responses though not significantly (*P* = 0.24) (Fig. 7A). In contrast, forskolin (1 μM) reduced mean tone by 48.62 ± 11.52% compared to vehicle control (*P* = 0.006) (Fig. 7B). The pre-addition of retigabine did not affect the response to isoprenaline. In contrast, XE991 attenuated the reduction of mean tone induced by isoprenaline and forskolin by 30.17 ± 18.07% (*P* = 0.13) and 26.24 ± 11.62% (*P* = 0.06), respectively, though not statistically significantly.

## Discussion

The present study has provided evidence that K_v_7 channels are functionally expressed in the human detrusor. RT-qPCR showed that all five subtypes of the *KCNQ* and *KCNE* genes, except for *KCNQ2*, were expressed in human bladder tissue. Functional studies confirmed the presence of functional K_v_7 channels in human detrusor. Furthermore, we observed a 3.4-fold up-regulation of *KCNQ1* in patients with bladder outflow obstruction compared to in patients with normal bladder function.

### Effect of K_v_7 channel modulators

Using the K_v_7.2–7.5 channel activator retigabine and the K_v_7 channel blocker XE991 we observed a potent effect of modulating K_v_7 channels in the human urinary bladder. Not only are K_v_7 channels functionally present in the human urinary bladder, they are also active at low level of depolarisation since XE991 augmented the pre-constriction tone achieved with 20 mM KPSS, but had no effect on the pre-constriction tone achieved with 40 mM KPSS- or 1 μM carbachol. This was also observed in pig detrusor [8] and could be due to phosphatidylinositol 4,5-biphosphate depletion as this has been shown to inactivate K_v_7 channels [38]. Mani et al. (2013) have shown that retigabine blocks L-type calcium channels and thus this could account for some of the effect mediated by retigabine, namely the fact that only retigabine, and not ML213, could decrease the contractility achieved with 20 mM KPSS [39]. However, in the paper by Rode et al. (2010) we tested the effect of retigabine on 5, 10 and 60 mM KPSS pre-constricted rat bladder tissue [10]. We found that retigabine concentration-dependently decreased the mean tone and twitch amplitude with a decrease in potency at increasing concentrations of K^+^. The effect was attenuated by the pre-incubation with XE991 (10 μM) and thus supports that the effect of retigabine in the present study is mediated by potassium channels. In guinea pig bladder, the application of meclofenamic acid has confirmed the presence of K_v_7.2 and/or K_v_7.3 channels [33] however functionality of K_v_7.3 channels remains to be studied in human urinary bladder.

In the present study on human urinary bladder, we show that *KCNQ1* is expressed and that the K_v_7.1 channel activator ML277 reduces tone in detrusor strips, however not at low concentrations of KPSS. In guinea pig bladder, K_v_7.1 channels could also be pharmacologically modulated by the blocker chromanol 293B [40] and activator L-364373 [11], though this was tested on spontaneous contracting bladder strips and thus did not completely correlate with our findings at near resting membrane potentials. Furthermore, using single cell electrophysiological recordings on freshly isolated guinea pig detrusor smooth muscle, functional K_v_7.1 channels (chromanol 293B) were observed by Anderson *et al*. in 2013 [40]. In contrast, we have formerly shown that chromanol 293B [8] and ML277 (S1 Fig.) had no effect on pig bladder and thus a complete overlap between human and pig bladder in terms of K_v_7 channel functionality is not evident.

Using myography, we showed that ML213 (positive K_v_7.2, K_v_7.4, K_v_7.4/7.5 and K_v_7.5 channel modulator) reduced mean tone of human tissue which correlated with our previous findings in pig urinary bladder [8]. We cannot with certainty claim that the effect is mediated via K_v_7.4 channels though this would be the most obvious as K_v_7.4 channels, at least on mRNA level, are more prevalent than the other K_v_7 channel subtypes. However, as protein levels of the channels have not been determined we do not know whether hetero- or homomeric K_v_7.4/K_v_7.5 channels exist in the urinary bladder. Though the functionality of K_v_7.4 or K_v_7.5 channels have not been studied in guinea pig bladder, Afeli *et al*. (2013) and Anderson *et al*. (2013) have performed immunohistochemical staining of K_v_7 channels [11]. Interestingly, while Afeli *et al*. (2013) claimed that K_v_7.4 channel protein could not be detected. Anderson *et al*. found protein of all K_v_7 channel subtypes in guinea pig bladder; which might be explained by different experimental settings.

### *KCNQ* and *KCNE* subtypes

The K_v_7 channels are formed by four α-subunits and modulated by two β-subunits encoded by the *KCNE* gene [41]. We observed that *KCNQ1* was 3.4-fold up-regulated in patients with bladder outflow obstruction compared to the patients with normal bladder function but there was no significant change in the level of either of the *KCNE* subtypes. However, *KCNE3* and *KCNE1* were 1.3-fold up-regulated (*P* = 0.38) and 1.9-fold down-regulated (*P* = 0.40), respectively. With regards to the other *KCNE* subtypes a slight change of 5–15% was observed between normal and BOO bladder biopsies. As K_v_7 channel activation leads to hyperpolarisation/repolarisation due to an outward current of K^+^, we expect that a higher level of K_v_7 channels will decrease the membrane potential and thus render the tissue with a lower baseline tension. It has been shown in cardiomyocytes that K_v_7.1-KCNE1 forms a slowly activating K^+^ current that is important for depolarisation. In the inner ear K_v_7.1-KCNE1 channels do not inactivate and as the channels are open at membrane potentials positive to -40 mV, the channels remain open [41]. Moreover, K_v_7.1 can be modulated by the ancillary KCNE3 subunit and KCNE3 has been shown to lock the open-state of the channel and render K_v_7.1 channels constitutively active thereby eliminating the channels voltage-dependency [42]. Therefore, we speculate that the up-regulation of the ancillary β subunits are shifting the channel from a voltage-dependent K_v_7.1-KCNE1 channel to a constitutively active K_v_7.1-KCNE3 channel and that this is a compensatory mechanism to avoid detrusor overactivity due to BOO to maintain normal bladder function. Though the findings in the present study are very interesting and calls for future studies, the number of studied patients with BOO is very low, and care must be taken not to overestimate the significance of the small changes in *KCNE* expression.

### Kv7 channels and the Gs pathway

The β3-adrenoceptor agonist mirabegron has been approved for the treatment of OAB. In the urinary bladder, β3-adrenoceptors are the most functionally important of the three characterised subtypes [43] and the mRNA level of β3-adrenoceptors have been shown to account for 97% of the total transcript level of β-adrenoceptors, both in patients with normal bladder function and in patients with BOO [44]. Studies on rat bladder has led to the hypothesis, that β3-adrenergic stimulation does not necessarily lead to cAMP production subsequent to adenylyl cyclase stimulation as Frazier *et al*. (2005) and Uchida *et al*. (2005) have shown that in strips pre-constricted by KCl, β3-adrenoceptor activation led to opening of big-conductance potassium channels (BK_Ca_), whereas relaxation of strips with passive tone was cAMP and thereby protein kinase A-dependent [45,46].

Moreover, it has been shown in rat renal arteries that β-adrenoceptor mediated relaxation relies on K_v_7.4 channel activation. Therefore, in addition to study K_v_7 channel function and expression, we also aimed to look at the possible interplay between K_v_7 channels and β-adrenoceptors in the human urinary bladder. More specifically we wanted to test the hypothesis that K_v_7 channels are important for β-adrenoceptor mediated relaxation. Firstly, we found that both isoprenaline and forskolin concentration-dependently decreased mean tone induced by pre-constriction with 1 μM carbachol-, 40 mM and 20 mM KPSS, with a slightly higher efficacy on strips pre-constricted with KPSS. Though we observed a time-dependent decrease in vehicle response at carbachol pre-constriction, these findings support previous studies on normal human urinary bladder strips [47]. We found that forskolin concentration-dependently relaxed human urinary bladder strips independent of the pre-constriction agent used. Forskolin acts by stimulating adenylyl cyclase and thereby increasing cAMP production, and therefore we believe that the cAMP pathway is intact in normal human urinary bladder strips. The cAMP pathway has also been shown to be intact in normal and hyperreflexic human urinary bladders by Igawa *et al*. (2001) [48]. To elucidate if the effect of isoprenaline was cAMP dependent we employed the protein kinase A inhibitor Rp-cAMPS (cell-permeable inhibitor of cAMP-dependent kinase A) [49], on pig urinary bladder, on both 1 μM carbachol and 40 mM and 20 mM KPSS-induced pre-constriction. The compound did not decrease the relaxation induced by isoprenaline (S2 Fig.). This is equivalent to findings made by Frazier *et al*. (2005) in rat bladder strips, in which H7 (protein kinase A inhibitor) failed to reduce contractions induced by 50 mM KPSS [45]. Interestingly, Frazier *et al*. (2005) showed that H7 decreased the potency of isoprenaline at passive tension. Therefore studies on passive tension should be performed on human tissue to determine if the cAMP pathway is active under these circumstances.

To evaluate the impact of K_v_7 channels on the Gs pathway, we pre-incubated the strips with retigabine or XE991 to activate and inhibit K_v_7 channels, respectively. In contrast to what we expected, K_v_7 channels were not affecting the isoprenaline- or forskolin-mediated relaxations at any pre-constriction agent tested. Therefore, it does not seem that β-adrenergic stimulation is *via* K_v_7 channels in the human urinary bladder. This means that the relaxation made by isoprenaline and retigabine is mediated by two separate mechanisms and proposes the use of K_v_7 channel activators as add-on therapy to β3-adrenergic agonists such as mirabegron.

### Clinical implications

The present study revealed an up-regulation of *KCNQ1* mRNA in patients with BOO. Furthermore, we now have pharmacological evidence that K_v_7.1 channels are functional in human bladder. Though this seems compelling for the development of positive modulators for K_v_7.1 channels for the treatment of urinary incontinence, it must be stated that K_v_7.1 channels are important in heart tissue [50,51] and therefore the risk of side effects renders K_v_7.1 channels as an improper target in the bladder. However, the urinary bladder biopsies also expressed *KCNQ4* and *KCNQ5* of which only *KCNQ4* has been described in heart tissue [52]. Accordingly, we would expect a lower degree of side effects when modulating these channels. This is in accordance with *in vivo* studies on rats in which retigabine increased micturition volume and interval at a lower dose, which had no effect on blood pressure [53] and further supported by clinical studies on retigabine in which urinary retention [9] was observed but no effects on the cardiovascular system was observed [54].

## Conclusions

K_v_7 channels are important regulators of urinary bladder quiescence at near resting membrane potential (-60 mV). We found that K_v_7.1 channels could be pharmacologically activated and that *KCNQ1* was up-regulated in patients with BOO. The performed gene expression analysis combined with the organ bath studies leads us to believe that compounds that activate K_v_7.4 and/or K_v_7.5 channels could be useful for treatment of overactive bladder syndrome.

## Supporting Information

S1 File The file describes the materials and methods used to study the effect of ML277 (Kv7.1 activator), chromanol 293B (Kv7.1 blocker) and rp-cAMPS (PKA inhibitor) on strips pre-constricted with 1 μM carbachol, 40 mM KPSS or 20 mM KPSS. All results are described in the results section corresponding to S1 and S2 Figs.
(DOCX)

S2 File The file contains all raw data that the study of Kv7 channels function has been based on. The naming of each sheet corresponds to the relevant figure or table.
(XLSX)

S1 Fig ML277 and chromanol does not affect mean tone of pig detrusor strips. ML277 (10 μM), chromanol (10 μM) or vehicle control (0.1% DMSO) was applied to strips pre-constricted by 1 μM carbachol (A), 40 mM KPSS (B) or 20 mM KPSS (C).
(TIF)

S2 Fig Inhibition of protein kinase A does not decrease the response to isoprenaline in pig detrusor strips. Rp-cAMPS (100 μM) or vehicle control (0.1% DMSO) was applied to strips pre-constricted by 1 μM carbachol (A), 40 mM KPSS (B) or 20 mM KPSS (C). Thereafter strips were treated with isoprenaline (0.1 μM).
(TIF)
