# Supplementary material for: Functional and Molecular Evidence for Kv7 Channel Subtypes in Human Detrusor from Patients with and without Bladder Outflow Obstruction
Source: PLoS One. 2015 Feb 18;10(2):e0117350. doi: 10.1371/journal.pone.0117350 (PMC4333569; doi:10.1371/journal.pone.0117350)
Supplement: S1 File — All results are described in the results section corresponding to S1 and S2 Figs. (DOCX) [file pone.0117350.s001.docx]

**Materials and methods S1 and S2**

*Tissue collection*

Danish landrace pigs (*n* = 11, 6–7 month-old, 105 kg) (The Danish Meat Trade College, Roskilde) were euthanised in accordance with the regulations and guidelines from the Danish Ministry of Food, Agriculture and Fisheries (DK-10-3-oth-036).

Detrusor specimens to be used for organ bath studies were kept in 4°C Tyrode’s salt solution (Sigma-Aldrich Denmark A/S, Broendby, Denmark) with 20mM HEPES (Life Technologies Europe B.V., Naerum, Denmark), (pH at 7.4) and transported immediately to the Smooth Muscle Research Center at Koege Hospital.

*Organ bath studies*

ML277 were kindly provided by Vanderbilt University (Nashville, TN, USA (MLCPN probes)). Chromanol 293B, papaverine and isoprenaline were from Sigma-Aldrich (St. Louis, MO, USA) whereas carbachol was purchased from Tocris (VWR international, Herlev, Denmark). All stock solutions (10^-2^ M), except for isoprenaline (dissolved in water), were prepared in 100 % DMSO (Merck, VWR international, Herlev, Denmark). Further dilutions were prepared in physiological saline solution (PSS) (NaCl 118.99 mM, KCl 4.69 mM, MgSO_4_•7H_2_O 2.40 mM, KH_2_PO_4_ 1.18 mM, glucose 6.06 mM, NaHCO_3_ 25 mM, CaCl_2_ 1.6 mM, EDTA 0.025 mM, pH 7.4) just before experiments.

Upon arrival the tissue was immediately placed in cold oxygenated PSS. The detrusor was separated from the urothelium under a stereomicroscope. The detrusor was then cut into strips, each 3 mm in length and 1 mm in width and kept on ice. The strips were then mounted in the organ bath (1 ml) of a myograph (700MO; Danish Myo Technology (DMT), Denmark), kept at 37°C and bubbled with a gas mixture of 95% oxygen and 5% carbon dioxide to a pH of 7.4 in PSS-buffer. The digital output was transformed using a powerlab (AD instruments, DMT, Aarhus, Denmark).

The strips were stretched by 2.5 mm during 30 minutes followed by an equilibration period of 60 minutes, in this period the bath solution was changed at 15 minutes intervals.

Detrusor strips were pre-constricted by 20 mM K-PSS, 40 mM K-PSS or 1 µM carbachol. When contractions were stable, pig detrusor strips were treated with ML277 (10 µM), chromanol 293B (10 µM) or vehicle control for 15 min. This allowed for the evaluation of K_v_7.1 channel mediated effects. To study the Gs pathway in pig detrusor the strips were pre-treated with rp-cAMPs (100 µM) or vehicle for 15 min. followed by the addition of isoprenaline (0.1µM). Maximal relaxation was induced by 100 µM papaverine whereas strips viability was assessed by stimulations of the detrusor strip with a buffer containing 125 mM K^+^ (KPSS). KPSS was similar to PSS except that NaCl was exchanged with KCl on an equimolar basis.

All values were normalised to the pre-constriction level before addition of compound. The mean tone was calculated, using LabChart Pro 7.3.7 software (AD instruments, DMT, Aarhus, Denmark), from an average of the last 2 minutes trace just before addition of the next concentration of compound. Graphic presentations were prepared using GraphPad Prism 5.04 (GraphPad Software, San Diego California, USA). Differences in mean tone between compound and vehicle control was analysed by unpaired *t*-test. The number of subjects is denoted by *n* and data are presented as mean values ± S.E.M.

**Results S1 and S2**

*K_v_7.1 channel functionality*

To study if K_v_7.1 channels are functional in pig urinary bladder we applied ML277 (10 µM) and chromanol 293B (10 µM) to strips pre-constricted by either carbachol (1µM) or KPSS (20 and 40 mM) (Figure S1). Activating K_v_7.1 channels by ML277 (1 µM carbachol, *P*=0.94, n=4; 40 mM KPSS, *P*=0.37, n=4; 20 mM KPSS, *P*=0.31, n=4) or inhibiting the channels by chromanol 293B (1 µM carbachol, *P*=0.65, n=5; 40 mM KPSS, *P*=0.15, n=4; 20 mM KPSS *P*=0.77 n=6) did not affect tone more than the application of vehicle control (1 µM carbachol, n=4; 40 mM KPSS, n=5; 20 mM KPSS, n=4).

*Protein kinase A dependency*

In pig detrusor strips we aimed at elucidating whether isoprenaline mediated relaxation is through protein kinase A and thus cAMP formation (Figure S2). Strips were pre-incubated with either rp-cAMPS (100 µM) or vehicle control (0.1% DMSO) before the application of isoprenaline (1 µM). At neither 1 µM carbachol (*P*=0.37), 40 mM KPSS (*P*=0.75) or 20 mM KPSS (*P*=0.56) pre-constriction, did the blockade of protein kinase A affect the response to isoprenaline.
