# Supplementary figures and images for: Functional and Molecular Evidence for Kv7 Channel Subtypes in Human Detrusor from Patients with and without Bladder Outflow Obstruction
Source: PLoS One. 2015 Feb 18;10(2):e0117350. doi: 10.1371/journal.pone.0117350 (PMC4333569; doi:10.1371/journal.pone.0117350)

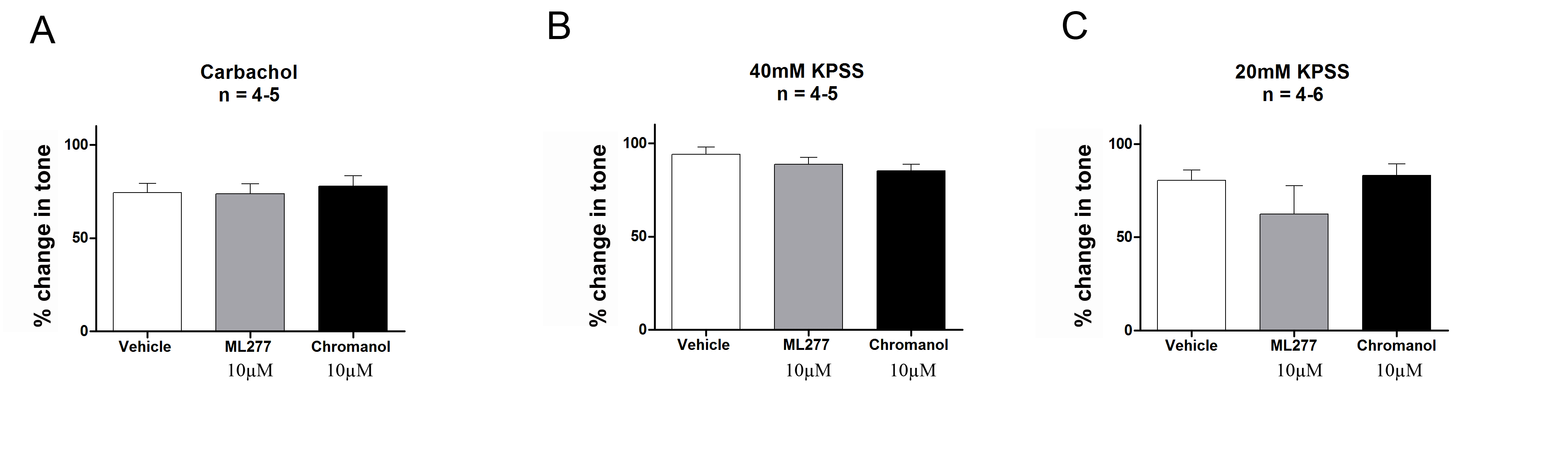

Supplement: S1 Fig — ML277 (10 μM), chromanol (10 μM) or vehicle control (0.1% DMSO) was applied to strips pre-constricted by 1 μM carbachol (A), 40 mM KPSS (B) or 20 mM KPSS (C). (TIF) [file pone.0117350.s003.tif]

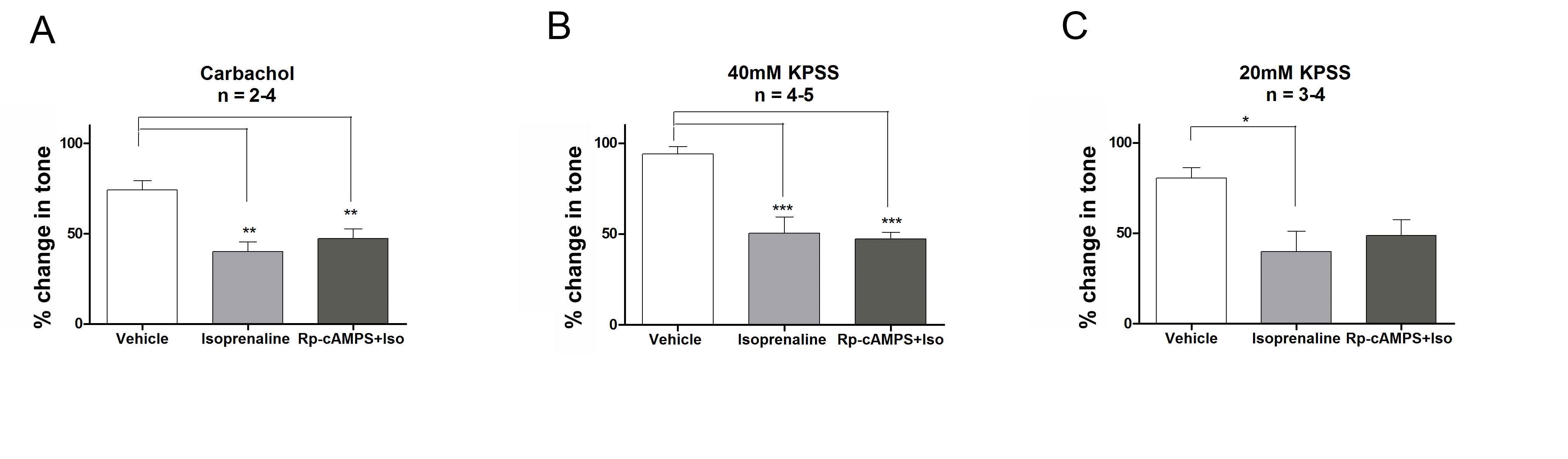

Supplement: S2 Fig — Rp-cAMPS (100 μM) or vehicle control (0.1% DMSO) was applied to strips pre-constricted by 1 μM carbachol (A), 40 mM KPSS (B) or 20 mM KPSS (C). Thereafter strips were treated with isoprenaline (0.1 μM). (TIF) [file pone.0117350.s004.tif]
